# Evaluation of Cytotoxic T Lymphocytes and Natural Killer Cell Distribution in Oral Squamous Cell Carcinoma and Oral Epithelial Dysplasia: An Immunohistochemical Study

**DOI:** 10.7759/cureus.56323

**Published:** 2024-03-17

**Authors:** Sneha John, Anna P Joseph, Varun B Raghavan Pillai, Pratibha Ramani, Jayanthi P, Karthikeyan Ramalingam

**Affiliations:** 1 Oral and Maxillofacial Pathology, PMS College of Dental Science and Research, Trivandrum, IND; 2 Oral and Maxillofacial Pathology, Saveetha Dental College and Hospital, Saveetha Institute of Medical and Technical Sciences, Saveetha University, Chennai, IND; 3 Oral Pathology and Microbiology, Saveetha Dental College and Hospital, Saveetha Institute of Medical and Technical Sciences, Saveetha University, Chennai, IND; 4 Oral Pathology, Azeezia Dental College, Kollam, IND

**Keywords:** immune cells, natural killer cell, t lymphocyte, expression, marker, immunohistochemistry, oral epithelial dysplasia, malignant transformation, oral leukoplakia, oral squamous cell carcinoma

## Abstract

Background

The tumor microenvironment comprises stromal cells, a few immune cells, vascular channels, and an extracellular matrix. The immune cells play a pivotal role in arresting the development of various tumors by identifying and killing the abnormal tumor cells. These immune cells with cytotoxic function include the natural killer (NK) cells and CD8+ T lymphocytes. Human NK cells express the cell surface marker CD57 and can be identified by using monoclonal antibodies. CD8+ cytotoxic T cells are a critical subpopulation of T cells and are important mediators of adaptive immunity. The anti-tumor immunity is important to assess the prognosis of tumors and develop new therapies. This study aimed to evaluate the immunohistochemical expression of CD8 and CD57 immune cells in oral squamous cell carcinoma (OSCC), oral epithelial dysplasia (OED), and normal oral mucosa.

Methodology

Clinically diagnosed and histopathologically confirmed cases of OSCC (n = 22), oral leukoplakia with OED (n = 22), and normal oral mucosa (n = 22) comprised the study groups. The tissue sections were subjected to immunohistochemical analysis for CD8 and CD57 expression by calculation of the mean labeling index. The results were statistically analyzed using a one-way analysis of variance, Bonferroni multiple comparison test, and Student’s t-test. SPSS software version 20.0 (IBM Corp., Armonk, NY, USA) was used for the statistical analysis, and the significance level was set at 0.05.

Results

An overall statistically significant difference was obtained in the number of CD8+ T lymphocyte cells and CD57+ NK cells when compared between OSCC, OED, and normal oral mucosa (p = 0.01). Variations in the number of CD8+ T lymphocyte cells and CD57+ NK cells were observed when a comparison was made between OED and OSCC and between OSCC and normal mucosal samples (p = 0.01). The study results showed that the mean labeling index of CD8 and CD57 increased in OSCC when compared to OED and normal mucosa (p = 0.01).

Conclusions

Samples of OED with moderate or severe dysplasia and samples of OSCC were accompanied by a higher level of infiltrating immune cells such as T cells, B cells, NK cells, and macrophages when compared to normal mucosa. The results suggested that the expression of CD8 and CD57 cells increased from normal mucosa to OED and the highest expression was found in OSCC. CD8 and CD57 could be used as surrogate markers to assess the malignant potential of the lesion and to determine the prognosis of patients with oral cancer.

## Introduction

In patients with head and neck cancer, oral squamous cell carcinoma (OSCC) is regarded as a significant cause of morbidity and mortality. The survival rate of patients with OSCC ranges between 45% and 50%, and survival has been reported to slightly improve with multimodal therapy. Such a low rate of survival in OSCC patients highlights the disease’s aggressiveness as well as the lack of appropriate knowledge about it, which impedes the development of potent treatments [1]. Oral cancer is considered to be the sixth to eighth most prevalent cancer worldwide. In India, the incidence of oral cancer in males and females is reported to be 8.7 and 3.5 per 100,000 population, respectively. Tobacco use is one of the main risk factors linked to oral cancer, which has a complex etiology [2,3]. In a majority of the instances, OSCC is preceded by oral potentially malignant diseases, the most frequent of which is leukoplakia [4]. The likelihood of leukoplakia developing into cancer, or the malignant transformation rate, varies between 5% and 18%, and the degree of epithelial dysplasia on histological examination is considered to be the primary factor determining the malignant transformation. Dysplastic features in the stratified squamous epithelium are characterized by cellular atypia, loss of normal maturation, and stratification. More than 20 classification systems have been proposed in the past 20 years for standardization of oral epithelial dysplasia (OED) grading. The 2017 World Health Organization (WHO) Criteria for Epithelial Dysplasia categorizes it to architectural changes and cellular changes [4,5].

The development of OSCC is a complex, heterogeneous process that depends on the interactions between genetically altered cells and their surrounding microenvironment [6]. For nutritional support and waste product disposal, the tumor stroma, also known as the tumor microenvironment, is necessary. The tumor microenvironment consists of blood vessels, innate and adaptive immune cells, and connective tissue. Adaptive immune responses are usually carried out by T and B lymphocytes, whereas the macrophages, dendritic cells, and natural killer (NK) cells are involved in innate immunity [7]. The immune cells recognize and eliminate changed or aberrant neoplastic cells, preventing the growth of different tumors. NK cells and CD8+ T lymphocytes identify and eliminate the immunogenic neoplastic cells in the early stages of tumor formation [8].

CD 8+ lymphocytes, the crucial T-cell subset, are the major groups of cells in the adaptive immune system. The major histocompatibility complex class I (MHC-I) receptor allows them to engage with and kill diseased or aberrant cells, making them a well-documented effector of immunity. Head and neck squamous cell carcinoma cells exhibit direct production of immunosuppressive chemicals such as transforming growth factor-beta and prostaglandin E2, as well as decreased MHC and co-stimulatory molecule expression, increased FasL expression, and decreased expression of co-stimulatory molecules. As a result of these changes, tumor-infiltrating CD8+ cells have a variety of functional flaws, including decreased Interleukin (IL) 2 production and lower proliferation, which can also aid in the tumor’s ability to persist [9,10]. By secreting cytokines or interacting directly with dendritic cells, NK cells are involved in both types of immune response. CD57 expression is noted on these NK cells indicating the maturative pathway. Through the release of the cytokine, interferon-gamma, and the eradication of perforin-dependent target cells, NK cells help tumor immunosurveillance and provide resistance to infections [7]. Thus, this study aimed to examine the expression of cytotoxic T lymphocyte and NK cells, two cell types that are crucial components of the tumor stroma in OSCC and leukoplakia samples exhibiting OED.

## Materials and methods

Sample collection

The sample size was calculated for this study using the formula n = Z^2^ P(1-P)/ d^2^, where n is the sample size, Z is the statistic corresponding to the level of confidence, P is the expected prevalence, and d is precision. The sample was calculated to be 22 for each group, incorporating values for the expected mean difference in immune cells between OSCC and OED, the effect size of 1, power (1-β) as 80%, and the level of significance (ɑ) as 5% [9].

The tissue sections of oral leukoplakia with OED (n = 22) and OSCC (n = 22) were taken from the archival blocks in the Department of Oral Pathology at PMS Dental College, Thiruvananthapuram. Biopsy specimens from both genders falling within the age range of 30-75 years were considered for this study. The tissue sections without epithelium were not considered for this particular study. For normal oral mucosa (n = 22), the tissue samples were taken during the surgical removal of impacted third molars. Individuals with any systemic illness or any known inflammatory conditions about the third molar were excluded. All specimens were used after obtaining written informed consent from all the participants. The study was reviewed and approved by the Institutional Ethics Committee of PMS Dental College (approval number: PMS/IEC/2020-21/02).

Immunohistochemistry

The tissue sections of 4 μm thickness were obtained from archival tissues and were studied for the expression of CD8 and CD57 by immunohistochemistry. The sections were placed in different grades of ethyl alcohol and then kept in distilled water. Subsequently, hydrogen peroxide was used to block the activity of endogenous peroxidase. Then, antigen retrieval with tris-ethylenediamine tetra-acetic acid buffer (pH 9.0) was performed. The tissue sections were incubated with primary antibody CD8 (Clone: C8/468, PathnSitu Biotechnologies Mouse Monoclonal Antibody CD8a, Telangana, India) and CD57 (Clone: NK1, PathnSitu Mouse Monoclonal Antibody CD57, Telangana, India). PathnSitu secondary kit and 3,3’ diaminobenzidine substrate solution were used, resulting in a colored precipitate at the specific sites of antigen binding. Hematoxylin was used as a counterstain, aiding in properly visualizing the sections.

CD8 and CD57 labeling index

All slides were scanned and analyzed for immunostaining at a magnification of 400× using a light microscope. Regardless of the staining intensity, cells showing brown stains within the cytoplasm were defined as positive. Tonsil tissue was used as positive immunohistochemical control. CD57+ NK cells and CD8+ T lymphocyte cells were analyzed quantitatively by a single trained examiner unaware of the clinicopathological data. For the OSCC specimen, positive staining was assessed in three anatomic compartments (tumor epithelium, connective tissue stroma, and advancing tumor margin). For OED and normal mucosa samples, two anatomic compartments (i.e., epithelium and connective tissue stroma) were evaluated. For every tissue sample, 10 different areas of the lesion were assessed with the help of an ocular grid. The mean number of CD8+ T lymphocytes and CD57+ NK cells were counted per microscopic field (40×) for each case. After obtaining the sum values of each field, the mean positive cells for each case were calculated using the mean labeling index.

Statistical analysis

Statistical analyses were done using SPSS version 20.0 (IBM Corp., Armonk, NY, USA). P-values <0.05 were considered statistically significant. A one-way analysis of variance test with a post hoc test (Bonferroni multiple comparisons) was used to compare quantitative variables among the groups.

## Results

A total of 66 samples were used for this study. The positive control group consisted of a sample of tonsil tissue. In contrast, the study groups consisted of 22 samples of OSCC, 22 samples of OED, and 22 samples of normal mucosa that were age and gender-matched with the cases. The biopsy specimens of OSCC and OED were taken from the buccal mucosa, tongue, gingiva, and alveolar mucosa, and the details are shown in Table 1.

**Table 1:** Distribution of anatomic sites of the study samples. OSCC = oral squamous cell carcinoma; OED = oral epithelial dysplasia

Anatomic site				
	Buccal mucosa	Tongue	Alveolar mucosa	Gingiva
OSCC (n = 22)	7	10	3	2
OED (n = 22)	9	7	4	2

The study involved different grades of OSCC, including nine cases of well-differentiated tumors, 22 cases of moderately differentiated, and two samples of poorly differentiated OSCC. Similarly, different grades of epithelial dysplasia were studied comprising mild dysplasia (n = 12), moderate dysplasia (n = 8), and severe dysplasia (n = 2). The participants in the study had a mean age of 61.4 years, 54.8 years, and 55.7 years in OSCC, OED, and normal mucosa, respectively. There was a male predilection at a ratio of 3:2 for OSCC and OED groups (Table 2).

**Table 2:** Age and gender distribution among the study groups. OSCC = oral squamous cell carcinoma; OED = oral epithelial dysplasia

	Mean age (years)	Number of males	Number of females
OSCC (n = 22)	61.4	14	8
OED (n = 22)	54.8	14	8
Normal mucosa (n = 22)	55.7	12	10

**Figure 1:**
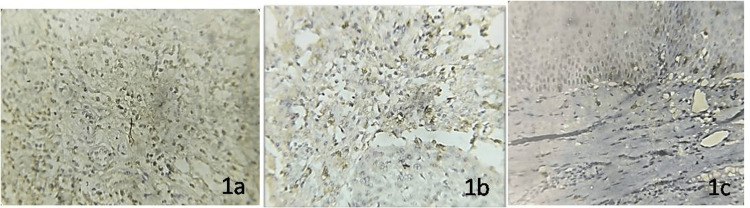
Immunohistochemical expression of CD8 in (a) oral squamous cell carcinoma, (b) oral epithelial dysplasia, and (c) normal oral mucosa.

The expression of CD8 and CD57 was the highest in OSCC, followed by OED, and low expression was found in normal mucosa. Figure 1 shows the immunohistochemical expression of CD8 and CD57 in the study groups.

**Figure 2:**
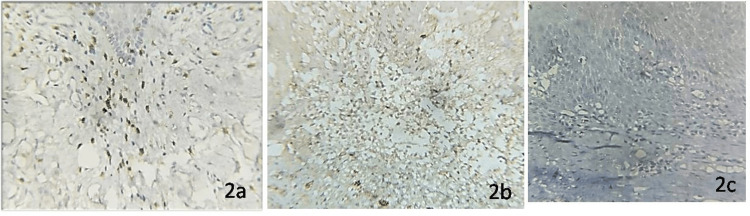
Immunohistochemical expression of CD57 in (a) oral squamous cell carcinoma, (b) oral epithelial dysplasia, and (c) normal oral mucosa.

Figure 2 shows the immunohistochemical expression of CD57 in the study groups.

Expression of CD8 and CD57 was primarily noticed within the inflammatory cells surrounding the tumor stroma of OSCC and sub-epithelial connective tissue of OED. An overall statistically significant difference was obtained in the number of CD8+ T lymphocyte cells and CD57+ NK cells when all three study groups were compared (p = 0.01). The study results showed that the mean labeling index of CD8 was 27.59 ± 12.71, 21.77 ± 7.99, and 6.40 ± 3.01 for OSCC, OED, and normal oral mucosa, respectively, and this difference was statistically significant (p = 0.01). OSCC samples exhibited a higher labeling index than OED and normal mucosa (p = 0.01) and OED samples had significantly higher labeling index when compared to normal oral mucosa (p = 0.01) (Table 3).

**Table 3:** Comparison of CD8 mean labeling index among the study groups. OSCC = oral squamous cell carcinoma; OED = oral epithelial dysplasia

	Mean (SD)	P-value
OSCC (n = 22)	27.59 (12.71)	0.01
OED (n = 22)	21.77 (7.99)	
Normal mucosa (n = 22)	6.40 (3.01)	

The mean labeling index of CD57 was 12.04 ± 7.69, 8.40 ± 4.99, and 3.21 ± 2.29 in OSCC, OED, and normal oral mucosa, respectively. A significantly higher labeling index was observed in OSCC samples compared to OED and normal mucosa (p = 0.01). Additionally, when OED and normal oral mucosa were compared, OED showed a higher labeling index. Table 3 and Table 4 show the comparison of the mean labeling index of CD8 and CD57 among the study groups.

**Table 4:** Comparison of CD57 mean labeling index among the study groups. OSCC = oral squamous cell carcinoma; OED = oral epithelial dysplasia

	Mean (SD)	P-value
OSCC (n = 22)	12.04 (7.69)	0.01
OED (n = 22)	8.40 (4.99)	
Normal mucosa (n = 22)	3.21 (2.29)	

## Discussion

The growth of tumors depends on the reciprocal interaction between the abnormal neoplastic cells and their microenvironment comprising immune cells, vascular channels, and extracellular matrix [6]. The tumor microenvironment is a complex entity that is actively involved in the progression and promotion of cancer [11]. T lymphocytes, dendritic cells, macrophages, and NK cells are the main types of immune cells observed within tumors [12]. As effective immunosurveillance is required to stop the formation and progression of cancer, numerous researchers have examined the relationships between the immune response and the development of malignant neoplasms. The immune system’s NK cells and CD8+ T lymphocytes are the cells most likely to be linked to a successful anti-tumor response. Pathogens and cancerous cells are largely eliminated by CD8+ cells, which are an essential part of cell-mediated immunity, whereas NK cells form a component of both innate and adaptive immunity. Virus-infected cells and tumor cells are the primary targets of CD8+ cytotoxic lymphocytes [13].

The highly matured NK cell has the most pronounced CD57 expression. The presence of NK cells that express CD57 is considered to be beneficial in cancer as these cells have a cytotoxic effect on tumor cells. NK cells form a major component of the tumor microenvironment and the expression of CD57 helps in evaluating the immune status of the patient [1]. The significance of immune cells in OSCC has been extensively studied. NK cells, an integral part of the innate immune mechanism, can kill tumor cells. These cells possess numerous receptors on their cell surface that have both activating and inhibiting properties. During homeostasis, the inhibitory receptors take a dominant role, and hence, the activity of NK cells is suppressed. However, when activating receptors are stimulated, NK cells exert cytotoxic effects and exhibit high levels of anti-tumor activity. Studies have shown that in cancers, the levels of activating receptors (NCR1, NCR2, NCR3) are decreased, and this finding has been linked to poor prognosis [14]. However, their role in oral premalignant lesions needs to be better elucidated in the literature. 

Our findings showed that there was a significant increase in the number of CD8+ T-lymphocyte cells in the connective tissue stroma of OSCC and OED when compared with other anatomic compartments. The individual intergroup comparison showed a statistically significant increase in the mean labeling index of CD8+ T-lymphocyte cells in OSCC (27.59 ± 12.71) and OED (21.77 ± 7.99) than normal mucosa (6.40 ± 3.01) with a mean difference of 21.18 and 15.36, respectively. A significant variation in the number of CD8+ T lymphocytes was noted between OED and normal mucosa, OSCC, and normal mucosa, as well as between OED and OSCC. These observations agree with the findings of Zancope et al. and Fang et al., who demonstrated a higher number of CD8+ T lymphocytes in OSCC compared with normal mucosa and premalignant lesions [1,13]. The higher number of CD8+ cells correlated with a lower mitotic index and a tendency toward a higher survival period [3].

Our study results showed a statistically significant increase in the mean value of the number of CD57+ NK cells in the connective tissue stroma of OSCC than OED and normal mucosa. The expression of CD57 was found to be the highest in OSCC, followed by OED and normal mucosa. A significant variation in the number of CD57+ NK cells was obtained between OED and OSCC, between OED and normal mucosal samples, and between OSCC and normal mucosa. Zancope et al. have shown a positive correlation between the overall survival rate and CD57+ NK cell counts in OSCC. Their study concluded that CD57 expression could be considered a powerful indicator of overall survival in patients with oral cancer [13].

Quality of life assessment is very critical for patients with OSCC [14]. Early diagnosis has a crucial role in determining the long-term prognosis. Researchers have demonstrated the presence of numerous NK cells and cytotoxic T lymphocytes in head and neck tumors. Increased numbers and dense infiltration of these immune cells are intended to kill abnormal tumor cells [15]. The use of immunohistochemical markers such as CD44, CD24, and NANOG in OSCC and OED has been correlated with survival and recurrence [16-18]. Metronomic therapy has been implemented for OSCC among Indian patients but its response or case selection could be influenced by the identification of relevant biomarkers or immunohistochemistry [16].

Santos et al. [19] have reported that higher CD8 expression was noted in T1 and T2 tumors of OSCC. Huang et al. [20] have emphasized the positive impact of CD57+ cell infiltration in clinical early-stage OSCC on overall survival. Ani et al. [21] have reported a reduction in the mean labeling index from well-differentiated OSCC to poorly differentiated OSCC. In our study, the expression of CD8 and CD57 was found to be higher in poorly and moderately differentiated tumors compared to well-differentiated OSCCs. However, this difference was not statistically significant. Similarly, the expression of CD8 and CD57 was higher in samples exhibiting moderate and severe dysplasia as compared to mild dysplasias.

The limitation of this study is that incisional biopsy specimens were used as study samples, and hence, it was difficult to assess the lymph node metastasis and proliferative index of the lesion. It is pivotal that future studies are performed using larger samples and regular follow-up of patients to provide a better understanding of the role of CD8+ T lymphocytes and CD57+ infiltrating immune cells and its impact on tumorigenesis, host immune response, and prognosis, thereby providing new clues to therapeutic strategies.

## Conclusions

The morbidity and mortality are variable among patients with oral cancer based on their tumor staging at the time of reporting. The tumor behavior, aggression, and recurring tendency are still not deciphered. Our results suggest that the expression of CD8 and CD57 cells increased from normal mucosa to OED and the highest expression was found in OSCC. Thus, CD8 and CD57 could be used as surrogate markers to assess the malignant potential of OED.

## References

[1] Fang J, Li X, Ma D (2017). Prognostic significance of tumor infiltrating immune cells in oral squamous cell carcinoma. BMC Cancer.

[2] Marocchio LS, Lima J, Sperandio FF, Corrêa L, de Sousa SO (2010). Oral squamous cell carcinoma: an analysis of 1,564 cases showing advances in early detection. J Oral Sci.

[3] Warnakulasuriya S (2010). Living with oral cancer: epidemiology with particular reference to prevalence and life-style changes that influence survival. Oral Oncol.

[4] Kaur P, Singh T, Kour S (2017). Malignant transformation of oral leukoplakia: an Asian scenario. J Adv Med Dent Sci Res.

[5] Warnakulasuriya S (2020). Oral potentially malignant disorders: a comprehensive review on clinical aspects and management. Oral Oncol.

[6] Tlsty TD, Coussens LM (2006). Tumor stroma and regulation of cancer development. Annu Rev Pathol.

[7] Agarwal R, Chaudhary M, Bohra S, Bajaj S (2016). Evaluation of natural killer cell (CD57) as a prognostic marker in oral squamous cell carcinoma: an immunohistochemistry study. J Oral Maxillofac Pathol.

[8] Teng MW, Galon J, Fridman WH, Smyth MJ (2015). From mice to humans: developments in cancer immunoediting. J Clin Invest.

[9] Maciel T, Serpa M, Mafra R, Gonzaga AKG, Souza LB, Pinto L (2017). Immunohistochemical analysis of natural killer cells and CD8+ T lymphocytes in lower lip squamous cell carcinoma. J Clin Diagn Res.

[10] Stelin S, Ramakrishan H, Talwar A, Arun KV, Kumar TS (2009). Immunohistological analysis of CD1a langerhans cells and CD57 natural killer cells in healthy and diseased human gingival tissue: a comparative study. J Indian Soc Periodontol.

[11] Anderson NM, Simon MC (2020). The tumor microenvironment. Curr Biol.

[12] Whiteside TL (2008). The tumor microenvironment and its role in promoting tumor growth. Oncogene.

[13] Zancope E, Costa NL, Junqueira-Kipnis AP (2010). Differential infiltration of CD8+ and NK cells in lip and oral cavity squamous cell carcinoma. J Oral Pathol Med.

[14] Ramalingam K, Krishnan M, Ramani P, Muthukrishnan A (2023). Quality of life assessment with European Organisation for Research and Treatment of Cancer Questionnaire (Head and Neck Module 43) and its clinicopathological correlation among patients treated for oral squamous cell carcinoma: an exploratory study. Cureus.

[15] Zhu X, Qin X, Wang X, Wang Y, Cao W, Zhang J, Chen W (2020). Oral cancer cell‑derived exosomes modulate natural killer cell activity by regulating the receptors on these cells. Int J Mol Med.

[16] Sethuraman S, Ramalingam K (2023). Metronomic chemotherapy in oral cancer: a review. Cureus.

[17] Poothakulath Krishnan R, Pandiar D, Ramani P, Ramalingam K, Jayaraman S (2023). Utility of CD44/CD24 in the outcome and prognosis of oral squamous cell carcinoma: a systematic review. Cureus.

[18] Arya I, Raghavan Pillai VB, Joseph A (2024). Identification and evaluation of cancer stem cells in oral squamous cell carcinoma and oral epithelial dysplasia using NANOG: an immunohistochemical study. Cureus.

[19] Santos EM, Rodrigues de Matos F, Freitas de Morais E, Galvão HC, de Almeida Freitas R (2019). Evaluation of Cd8+ and natural killer cells defense in oral and oropharyngeal squamous cell carcinoma. J Craniomaxillofac Surg.

[20] Huang Z, Lu Y, Wang W (2023). Prognostic value of tumor-infiltrating immune cells in clinical early-stage oral squamous cell carcinoma. J Oral Pathol Med.

[21] Cs AS, Joseph TI, Girish KL, T P, Binu A, Mary J (2023). Comparative analysis of cluster of differentiation 57 and proliferating cell nuclear antigen expression in different grades of oral squamous cell carcinoma: an immunohistochemical study. Cureus.

